# A Novel Glucosamine-Based Cannabidiol Complex Based on Intermolecular Bonding with Improved Water Solubility

**DOI:** 10.3390/molecules30153179

**Published:** 2025-07-29

**Authors:** Mitja Križman, Jure Zekič, Primož Šket, Alojz Anžlovar, Barbara Zupančič, Jože Grdadolnik

**Affiliations:** 1 National Institute of Chemistry, Hajdrihova 19, 1000 Ljubljana, Slovenia; primoz.sket@ki.si (P.Š.); alojz.anzlovar@ki.si (A.A.); barbara.zupancic@ki.si (B.Z.); joze.grdadolnik@ki.si (J.G.); 2 Faculty of Maritime Studies and Transport, University of Ljubljana, Pot pomorščakov 4, 6320 Portorož, Slovenia

**Keywords:** cannabidiol, glucosamine, cannabinoids, water solubility, bioavailability

## Abstract

In this study, a new, patented form of a water-soluble cannabidiol (CBD) complex was synthesised and tested. The formation of the complex is based on the interactions, presumably through hydrogen bonding, between cannabidiol and glucosamine, the latter contributing significantly to the increased hydrophilicity. The complex was characterised by chromatography, thermal analysis, nuclear magnetic resonance, Fourier transform infrared spectroscopy, and permeability tests. This complex has a substantially higher water solubility than normal CBD. Permeability tests indicate that it has almost five times lower permeability through lipophilic membranes and less than half the membrane mass retention of conventional CBD. At the same time, its equilibrium concentration is almost four times higher than that of normal CBD. These results suggest that this new form of CBD is a promising candidate for future biological and clinical studies, as it offers improved bioavailability and biodistribution.

## 1. Introduction

Cannabidiol (CBD), the main non-psychoactive cannabinoid extracted from *Cannabis sativa*, has garnered significant interest in oncology due to its potential to complement conventional chemotherapeutic regimens. Extensive research suggests that CBD can induce apoptosis, inhibit cancer cell proliferation, and reduce metastasis across various cancer models, including breast, lung, colon, and prostate cancers [1,2]. Furthermore, CBD has been shown to enhance the efficacy of standard chemotherapeutic agents while simultaneously reducing their toxic side effects, positioning it as a promising adjuvant in cancer therapy [3,4]. Beyond oncology, CBD has demonstrated potential therapeutic benefits for other medical conditions, including diabetes, hyperglycaemia [5], and various dermatological disorders [6].

One of the major limitations of CBD is its low water solubility, which leads to poor oral bioavailability. To address this challenge, water-soluble CBD formulations have been developed to improve absorption and enhance therapeutic efficacy. In studies involving colorectal cancer cells, water-soluble CBD formulations have been shown to induce apoptosis at a significantly higher rate than traditional lipid-soluble CBD, with a 55% increase in apoptotic cells [7]. Similarly, in breast cancer models, water-soluble CBD has proven effective in targeting cancer stem cells, which play a critical role in cancer recurrence and metastasis [1].

Various strategies have been employed to improve the water solubility of cannabinoids. The widely used term “nanoformulation” encompasses a range of CBD formulations based on polymers, lipid nanocapsules, nanoemulsions, cyclodextrins, and more. These nanoformulations offer improved pharmacokinetics and enhanced targeted delivery [8]. For instance, nanoemulsion technology—which disperses CBD into nanoscale droplets—significantly increases bioavailability due to the expanded surface area for absorption in the gastrointestinal tract [9]. Liposomal CBD formulations have also demonstrated superior cytotoxicity against multiple cancer cell types compared to non-liposomal CBD, further supporting the potential of advanced drug delivery systems [10]. In triple-negative breast cancer cells, nanoformulated water-soluble CBD has resulted in a 70% increase in apoptosis and a marked reduction in cell proliferation [11]. These innovations in CBD delivery could be pivotal in optimising its use as an adjuvant therapy, especially for hard-to-treat cancers. Another promising strategy involves the use of cyclodextrin-based carriers, which encapsulate CBD to form inclusion complexes [12]. These carriers enhance the solubility of hydrophobic compounds by enclosing them within a hydrophilic cavity. Studies have shown that CBD–cyclodextrin complexes yield significantly higher plasma concentrations compared to unformulated CBD when administered orally [13]. This approach not only improves bioavailability but also extends the therapeutic window, making it suitable for chronic treatment. Other methods for enhancing CBD water solubility include co-dispersion with silica particles or hydrophilic polymers such as polyvinylpyrrolidone, which also significantly boost solubility [14,15]. Additionally, complexation with phospholipids has been explored as a means of improving both solubility and overall biochemical performance [16]. In connection with the increasing water solubility and bioavailability of CBD, nanocarriers appear to be the predominant technical trend in recent years. Nanocarrier-based solutions utilise a variety of ingredients: synthetic, inorganic, and natural or naturally derived [17]. Some of these formulations consist only of natural ingredients [18,19,20]. However, many of these formulations contain at least one synthetic ingredient such as a polymer or a surfactant [21,22,23], resulting in a higher CBD content.

To achieve better bioavailability, we used a completely different strategy to improve the water solubility of CBD. We also prioritised the use of natural or naturally derived ingredients. This way, there are fewer opportunities to introduce ingredients that could potentially raise questions about safety. CBD has two hydroxyl groups attached to the phenol ring and is therefore a compound with an acidic character. We postulated the formation of a complex with higher water solubility when an acid–base interaction between CBD and a hydrophilic base occurs. With the aim of finding a highly hydrophilic substance with a basic character of natural origin, we quickly narrowed down the choice to amino sugars. Without counter anions (e.g., chloride, sulphate), amino sugars act as organic bases. There are several naturally occurring amino sugars, but the most common and probably the only cost-effective amino sugar is glucosamine. Glucosamine is an amino sugar that is usually obtained by the acid hydrolysis of chitin. Chitin is a natural biopolymer that is abundant in the exoskeletons of crustaceans, for example, and has already found application in many areas [24,25]. Therefore, we considered glucosamine to be the best practical and “green” solution to increase the water solubility of CBD using the acid–base interaction approach by complexing it with glucosamine [26].

The aim of this study was to characterise the resulting water-soluble CBD-GA complex using a range of physical, chemical, and biochemical techniques. The solubility, cell membrane permeability, and overall therapeutic potential were analysed and evaluated.

## 2. Results and Discussion

### 2.1. Physical and Spectroscopic Observations

**Figure 1:**
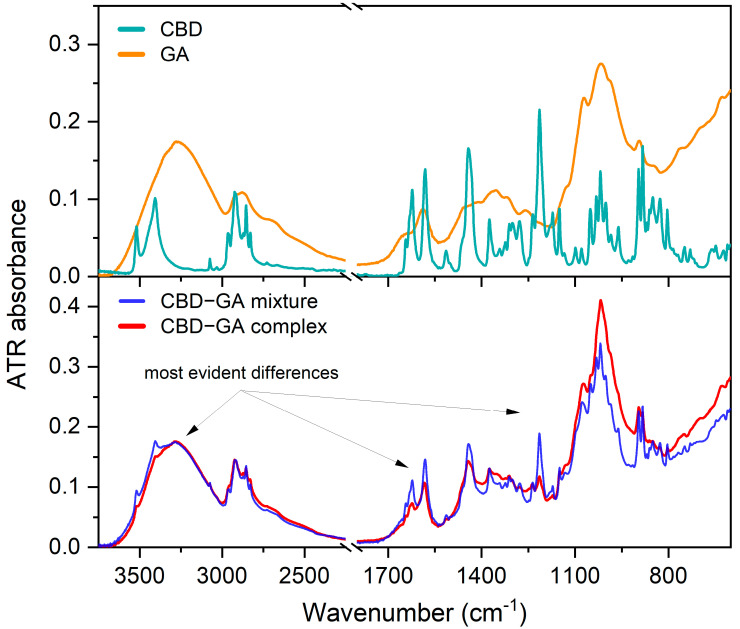
Measured ATR FTIR spectra. Upper diagram—ATR-FTIR spectra of CBD and GA compounds. Lower diagram—ATR-FTIR spectra of CBD-GA mixture and CBD-GA complex, both at molar ratio 1:2.

ATR-FTIR spectra were obtained for pure cannabidiol (CBD), glucosamine (GA), their physical mixture (CBD-GA mixture prepared without the complexation procedure), and the synthesised complex (CBD-GA complex). The spectra of the CBD and GA compounds are shown in the upper diagram of Figure 1, while the spectra of the CBD-GA mixture and the CBD-GA complex are shown in the lower diagram of Figure 1. The wavenumbers of the most intense bands in these spectra can be found in Appendix A.1.

**Figure 2:**
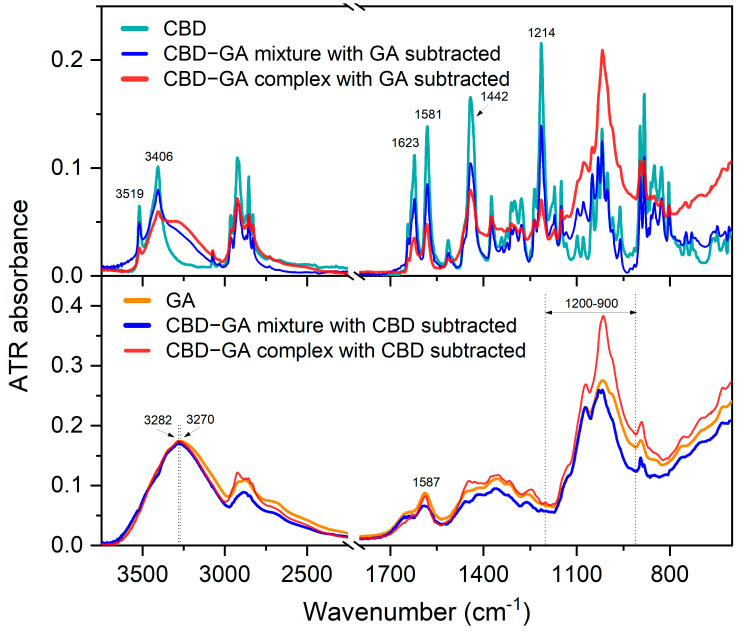
Difference ATR FTIR spectra. Upper diagram—ATR-FTIR spectrum of CBD compound compared to difference ATR-FTIR spectra of CBD-GA mixture and CBD-GA complex (both at molar ratio 1:2) after subtraction of GA compound spectrum. Lower diagram—ATR-FTIR spectrum of GA compound compared to difference ATR-FTIR spectra of CBD-GA mixture and CBD-GA complex (both at molar ratio 1:2) after subtraction of CBD compound spectrum.

At first glance, the physical mixture (blue trace in the lower diagram of Figure 1) shows narrower and more intense bands attributed to CBD than the CBD-GA complex (red trace in the lower diagram of Figure 1), while in the range between 1200 and 900 cm^−1^, the bands attributed to GA are more intense for the CBD-GA complex than for the CBD-GA mixture. To gain further insight, we calculated difference spectra by subtracting the GA spectrum from the spectra of the mixture and the complex and subtracting the CBD spectrum at another time. The obtained difference spectra compared to the spectra of the compounds are shown in Figure 2.

The difference spectra in the upper diagram of Figure 2 show that the CBD bands from the mixture remaining after the subtraction of the spectrum of the GA compound are similar in width to those of the CBD compound itself. On the other hand, the CBD bands from the complex that remained after subtracting the spectrum of the GA compound become broader. In particular, the broader OH stretching bands at 3519 and 3406 cm^−1^ [27,28] of CBD in the complex indicate an altered hydrogen bonding environment compared to the mixture and the CBD compound. The changes in the molecular interaction environment are also reflected in the broader bands at 1623, 1581, and 1442 cm^−1^ for the CBD-GA complex, which correspond to the vibrations of the benzene skeleton. A 2 cm^−1^ blue shift in the band at 1581 cm^−1^ assigned to =C-H [28,29] is observed in the case of the complex compared to the mixture and the CBD compound. The bands in the range between 1250 and 1230 cm^−1^, presumably assigned to the C-C twisting of the rings [30], are broader in the complex than in the mixture. Interestingly, the width of the band at 1214 cm^−1^, which is assigned to C-O stretching [29], remains similar to the corresponding CBD band in both the mixture and the complex.

The difference spectra in the lower diagram of Figure 2 reveal the following differences in the GA bands. The OH stretching band of GA at 3270 cm^−1^ [31] narrows and blueshifts by about 12 cm^−1^ in a similar way for the mixture and the complex. The N-H bending band at 1587 cm^−1^ becomes broader in the mixture and narrower in the complex than the corresponding band of the GA compound. The bands in the 1200–900 cm^−1^ range, which are attributed to the stretching of the glycosidic linkage (C–O–C) and the C–OH [31] in the sugar ring, are narrower in the mixture and even narrower in the complex.

In summary, the observed changes in the FTIR spectrum show the increased molecular conformational freedom of CBD and a decreased one for GA within the CBD-GA complex compared to the mixture. This is evidence of complexation-induced modifications in intra- and intermolecular interactions, which appear to be reflected in improved solubility.

**Figure 3:**
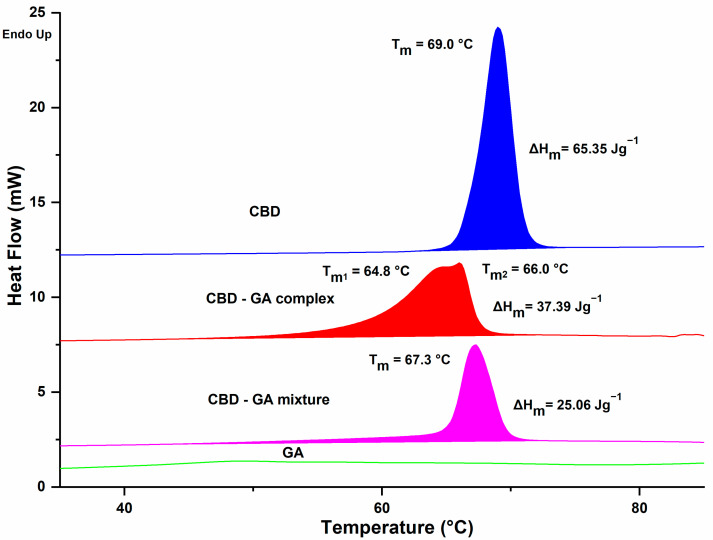
DSC analysis data. The corresponding compounds, their melting points, and melting enthalpies are indicated above each trace.

The DSC analysis revealed clear differences between the analysed samples (Figure 3). While virtually no observable changes were detected for GA within the measurement range (i.e., up to 110 °C), the observed phase change properties of CBD showed an almost symmetrical peak with a melting point of 69.0 °C and a melting enthalpy of 65.4 J/g. For the physical mixture CBD-GA, similar values for melting temperature and melting enthalpy to those for CBD were expected, but a decrease of about 1.7 °C was observed, with a less symmetrical peak. The expected enthalpy of melting of the mixture should be about 30 J/g due to the 47% mass content of CBD in the mixture (at a CBD:GA molar ratio of 1:2). However, the enthalpy was about 20% lower than the predicted value. A possible explanation for the behaviour of the CBD-GA mixture could be the formation of a eutectic mixture during the melting process, although the differences are not as pronounced as in the case of ascorbic acid and sugar [32], for example. In contrast to the mixture, the CBD-GA complex showed an even lower melting point around 3.0 °C compared to pure CBD but with a 50% higher enthalpy value compared to the CBD-GA mixture. This clearly indicates the existence of intermolecular CBD-GA interactions in the complex, which is consistent with the observations of the FTIR analysis. These interactions are most likely due to hydrogen bonding, which may even lead to the formation of co-crystals [33,34]. The melting behaviour of the complex shows a very asymmetric, overlapping double peak, which could indicate some heterogeneity or multiple structural domains within the complex (in the solid state). However, further studies using complementary techniques would be required to substantiate this hypothesis.

^1^H NMR spectra were recorded for the individual pure compounds and for the water-soluble CBD-GA complex (Appendix A.2). A physical mixture of CBD and GA was not analysed, as this experiment was conducted in solution, where intermolecular interactions between CBD and basic GA naturally occur—this being the fundamental mechanism of complex formation. It should also be noted that the ^1^H NMR spectrum of GA in its basic form was not suitable for detailed comparison due to the large number of peaks arising from its structural characteristics. As a monosaccharide, GA exists in multiple isomeric forms in solution, which results in a complex and highly congested spectrum. However, this limitation did not significantly hinder the analysis, as meaningful comparisons could still be made based on the well-defined ^1^H NMR spectrum of CBD. The observed spectral shifts and changes confirm the formation of interactions between CBD and GA (Table 1).

**Figure 4:**
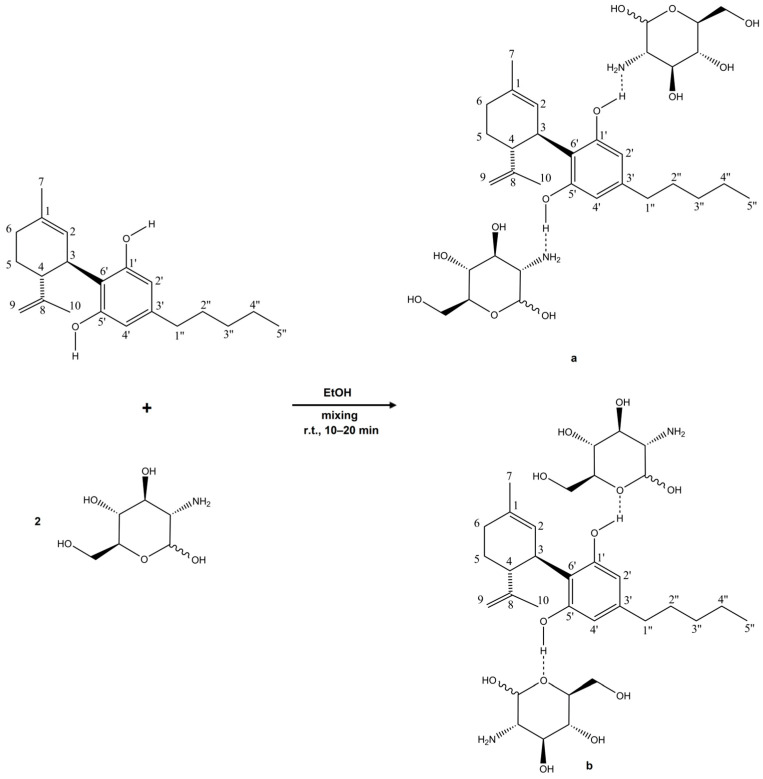
The reaction scheme and hypothetical structures of the complex between CBD and glucosamine. Based on the measurements, it can be concluded that interactions occur between the hydroxyl groups of CBD and the –NH_2_ groups (**a**) and the C–O–C groups (**b**) of glucosamine. The dotted lines represent putative hydrogen bonding.

The results of the NMR measurements for the CBD–GA complex indicate interactions involving the protons of the hydroxyl groups (–OH) of CBD, particularly at the 5′ and 1′ carbon atoms. In conjunction with the observations from the FTIR spectra, it is very likely that these hydroxyl protons interact with the C-O-C group fragment of GA, at least when the CBD-GA complex is in the solid (i.e., non-solubilised) state. This interaction is as expected, since hydrogen bonding usually occurs between the protons of the hydroxyl group (which have a strongly polar O–H bond and a resulting partial positive charge of the hydrogen atom) and electronegative oxygen atoms [35]. The presence of such possible interactions is confirmed by the observed changes in the NMR spectrum—in particular by an increase in the chemical shift in the hydroxyl proton signals and the broadening of the corresponding peaks. However, other types of interactions cannot be excluded when the CBD-GA complex is dissolved in an aqueous medium. Another possible interaction of CBD hydroxyl groups would also be with the -NH_2_ groups of GA, whereby the reorientation and (de)protonation of the molecules involved are plausible. Regardless of the actual intermolecular interactions, these spectral features confirm the presence of hydrogen bonding and consequently the formation of a complex between CBD and GA. The reaction scheme and the putative structures, which are based on intermolecular interactions, are shown in Figure 4.

### 2.2. Water Solubility

The water solubility values of pure CBD and its complexes with GA are presented in Table 2. All complex forms, across different molar ratios, exhibited an approximate 350- to 460-fold increase in solubility compared to pure CBD. Based on these results, the complex with a 1:2 molar ratio was selected for all subsequent experiments, as the difference in solubility between the 1:2 and 1:3 complexes was minimal.

The drastic increase in the water solubility of the complex is likely due to the high degree of hydration of the GA molecules, which probably effectively shield the CBD moiety between the GA units, preventing the precipitation of CBD. Pure glucosamine has been reported to have a high degree of hydration in an aqueous medium. Its first hydration layer consists of five to nine water molecules [36], which is even higher than that for glucose [37]. This can also explain the dramatic increase in the water solubility of the CBD-GA complex with a low molar ratio of only 1:1.

### 2.3. Permeability Analysis

**Figure 5:**
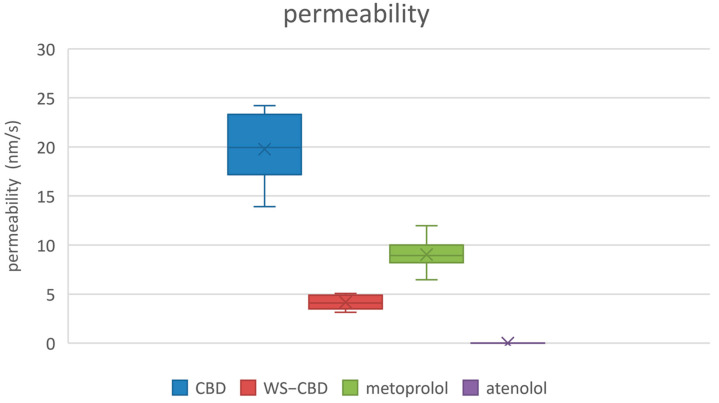
Measured permeabilities of studied substances. CBD—regular cannabidiol; WS-CBD—water-soluble cannabidiol (CBD:GA molar ratio 1:2).

**Figure 6:**
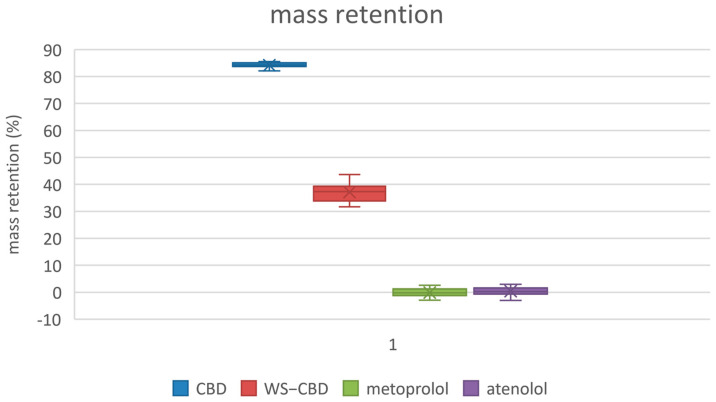
Membrane mass retention of substances. CBD—regular cannabidiol; WS-CBD—water-soluble cannabidiol (CBD:GA molar ratio 1:2).

**Figure 7:**
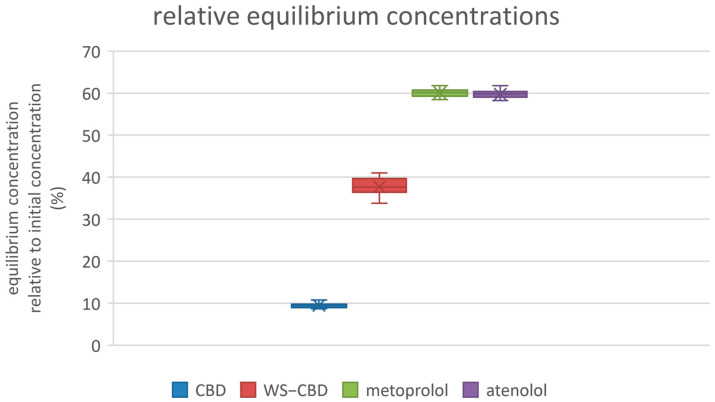
Relative equilibrium concentrations of substances. CBD—regular cannabidiol; WS−CBD—water-soluble cannabidiol (CBD:GA molar ratio 1:2).

The permeation tests revealed significant differences between regular CBD and the water-soluble CBD complex. As expected, the permeability of water-soluble CBD was approximately five times lower than that of regular CBD. The average permeabilities were 19.8 nm/s for regular CBD and 4.2 nm/s for the water-soluble form. Additionally, water-soluble CBD exhibited substantially lower mass retention in the lipophilic membrane—about 37% on average—compared to the 84% retention observed for regular CBD. More pronounced differences were also observed in the calculated equilibrium concentrations: regular CBD reached an equilibrium concentration of approximately 15.1 µM (9.5% relative concentration), which is about four times lower than the 60.1 µM (37.7% relative concentration) recorded for water-soluble CBD. The detailed results of the permeation tests are illustrated in Figure 5, Figure 6 and Figure 7.

The permeation test results indicate that water-soluble CBD has approximately five times lower permeability compared to regular CBD. However, it also exhibits significantly lower mass retention on the membrane and a higher equilibrium concentration. Combined with its markedly greater solubility—over two orders of magnitude higher—these factors suggest that water-soluble CBD is likely to have substantially improved bioavailability and cellular distribution compared to regular CBD. These permeability data can also be related to the same cause as discussed in Section 2.2, i.e., the large degree of hydration of GA and consequently to the complex itself. Probably being large in size with a large envelope of water molecules around it, the complex is less likely to permeate easily or to be retained in a lipophilic membrane. On the contrary, a higher equilibrium concentration can thus be more easily attained in an aqueous medium, probably for the same underlying reason.

## 3. Materials and Methods

### 3.1. Water-Soluble Complex Synthesis

GA in its free base form was prepared by desalting GA hydrochloride (Sigma-Aldrich, St. Louis, MO, USA) prior to the synthesis of the CBD complex. In a typical experiment, 5 g of GA hydrochloride was dissolved in 50 mL of water, and the resulting solution was passed dropwise through a glass gravity column (250 mL volume) packed with 50 g of Ambersep 900 anion exchange resin in the hydroxide (-OH) form (Sigma-Aldrich). The resin was pre-equilibrated with water. Elution was carried out using 10 column volumes of water. The eluates were collected, frozen at −80 °C, and subsequently freeze-dried. The absence of chloride ions was confirmed chromatographically using a Vanquish HPLC system (Thermo Scientific, Waltham, MA, USA) equipped with a CAD detector, following the method described by Dolci et al. [38]. See Appendix A.3 for details.

CBD-GA complexes were prepared at varying molar ratios of cannabidiol (CBD; Pharmahemp, Ljubljana, Slovenia) to GA base: 1:1, 1:2, and 1:3. In a representative 1:2 molar ratio experiment, 31.4 mg (0.1 mmol) of CBD was dissolved in 4 mL of ethanol (Carlo Erba, Milan, Italy), and 35.8 mg (0.2 mmol) of GA base was dissolved separately in 4 mL of ethanol. For other molar ratios, the amount of GA was adjusted accordingly. The GA solution was then added dropwise to the CBD solution under constant stirring with a magnetic stirrer. After complete addition, stirring was continued for an additional 10 min at room temperature.

Following the reaction, the solvent was evaporated under reduced pressure. The resulting product was used for water solubility and permeability tests and for characterisation via NMR, FTIR, and differential scanning calorimetry (DSC).

### 3.2. Water Solubility Analysis

The prepared CBD-GA complexes, in various molar ratios, were tested for water solubility. In a typical experiment, between 1.0 and 1.5 mg of the complex was accurately weighed and dissolved in 1 mL of deionised water (Merck Millipore, Burlington, MA, USA) under vigorous mixing. If necessary, an ultrasonic bath was employed to aid dissolution.

Following mixing, the solution was centrifuged for 3 min at 3000× *g*. The resulting supernatant was transferred to vials for HPLC analysis. The concentration of dissolved CBD was quantified using a chromatographic method [39] with an Agilent 1260 Infinity HPLC system (Waldbronn, Germany). Further details are provided in Appendix A.4.

### 3.3. Differential Scanning Calorimetry

Differential scanning calorimetry (DSC) analyses were performed using a DSC-1 instrument (Mettler Toledo, Greifensee, Switzerland). Measurements were conducted over a temperature range of 25 °C to 110 °C under an inert nitrogen atmosphere (flow rate: 20 mL/min). Samples were heated at a rate of 10 °C/min. Both physical CBD-GA mixtures and CBD-GA complexes as well as neat CBD and GA were analysed under these experimental conditions.

### 3.4. Fourier Transform Infrared Spectroscopy

Attenuated Total Reflectance Fourier Transform Infrared (ATR-FTIR) spectroscopy was performed using a Bruker Tensor 27 spectrometer (Bruker Optics, Billerica, MA, USA) equipped with a single-reflection Specac Golden Gate diamond ATR accessory and a liquid nitrogen-cooled mercury cadmium telluride (MCT) detector. All measurements were conducted at 298 K. To minimise atmospheric interference, the spectrometer’s optical path and the ATR sampling module were sealed and continuously purged with dry nitrogen gas during data acquisition.

Infrared spectra were collected over the spectral range of 4000–600 cm^−1^, with each spectrum obtained by co-adding 128 interferograms at a nominal spectral resolution of 2 cm^−1^. The temperature of the ATR assembly was precisely regulated using a Specac Heated Golden Gate temperature controller. Prior to each measurement, the diamond crystal surface was thoroughly cleaned with analytical-grade acetone to eliminate potential cross-contamination between samples.

Spectral contributions from ambient water vapour and carbon dioxide were monitored and mathematically corrected during post-processing to ensure spectral fidelity. Consistent with differential scanning calorimetry (DSC) protocols, both physical mixtures and supramolecular complexes were analysed under identical ATR-FTIR experimental conditions.

### 3.5. Nuclear Magnetic Resonance Spectroscopy

NMR analysis was also used to evaluate the water-soluble complex. A Bruker AVANCE NEO 400 MHz NMR spectrometer, with a 5 mm BB(F)O probe, was used for this purpose. Two to seven milligrams of CBD or complex was dissolved in 600 μL of DMSO-d6 (Sigma Aldrich). ^1^H NMR spectra were recorded using a zg pulse sequence at 25 °C. A total of 128 scans were taken, with a repetition delay of 3 s. The spectrum width was 8.6 kHz. Chemical shifts were calibrated against the DMSO signal. ^1^H-^13^C HSQC spectra were used for the assignment of individual signals.

### 3.6. Permeability Tests

In vitro permeation (PAMPA) tests were performed to evaluate differences in permeation parameters between regular (lipophilic) CBD and the water-soluble CBD complex. Atenolol and metoprolol were included as control substances. The initial concentration of CBD in the donor medium was 159 µM, while the concentrations of atenolol and metoprolol were 500 µM each. The experiments were conducted using a 96-well PAMPA plate with PVDF filter membranes (Sigma-Aldrich). The donor medium consisted of 50 mM phosphate buffer (pH 6.5, Sigma-Aldrich) containing 5% (*v*/*v*) DMSO, with a volume of 300 µL per well. The acceptor medium used was Pion’s “acceptor sink buffer” (Billerica, MA, USA), added in a volume of 200 µL per well. The artificial membrane solution consisted of 1% (*w*/*v*) cholesterol (Merck, Darmstadt, Germany) dissolved in a 3:1 (*v*/*v*) mixture of dodecane and *n*-hexane. Prior to the experiment, 4 µL of this membrane solution was applied to each filter membrane. Each test compound was assessed in 12 replicates. The total duration of the experiment was 260 min. The inclusion of DMSO in the donor medium was essential due to the extremely low aqueous solubility of unformulated CBD (less than 60 ng/mL) [14]—more than 400 times lower than that of the water-soluble CBD complex—making accurate quantification otherwise unfeasible. The low proportion of DMSO was sufficient to solubilise regular CBD without compromising the stability of the water-soluble complex. At the end of this experiment, both donor and acceptor solutions were analysed by HPLC. CBD concentrations were determined using HPLC with UV detection, according to the method described by Križman [39] (see Appendix A.4). Atenolol and metoprolol concentrations were analysed by HPLC with FL detection, following the protocol of Yilmaz et al. [40] (see Appendix A.5).

## 4. Conclusions

A new water-soluble form of CBD was synthesised and characterised using spectroscopic, chromatographic, and permeability methods. The data support the hypothesis of complex formation by hydrogen bonding between cannabidiol and glucosamine, which shows significantly improved water solubility. Further studies using complementary techniques are required for a more detailed investigation of the complex structure. The permeability study showed a markedly different behaviour compared to normal CBD, indicating significantly improved performance in aqueous media. Consequently, improved bioavailability is expected and will be evaluated in subsequent studies based on these results.

## Figures and Tables

**Table 1:** NMR data for pure and modified CBD (complex with glucosamine at CBD-GA ratio 1:2). Proton numbering refers to structure of CBD molecule shown in Figure 4 in hypothesised complex form. * indicates that signals overlap with signals from GA.

H	Multiplicity (Pure CBD)	δ/ppm (Pure CBD)	Multiplicity (Complex)	δ/ppm (Complex)
**2 (1H)**	s	5.07	s	5.07
**3 (1H)**	m	3.87–3.77	/	/ *
**4 (1H)**	m	3.02	/	/ *
**5 (2H)**	m	1.66	m	1.66
**6 (1H)**	m	1.91	m	1.91
**6 (1H)**	m	2.16–2.02	/	/ *
**7 (3H)**	s	1.59	s	1.59
**9 (1H)**	dd	4.39	dd	4.39
**9 (1H)**	d	4.48	d	4.48
**10 (3H)**	s	1.57	s	1.57
**2′,4′(2H)**	s	6.01	s	6.01
**1′,5′OH (2H)**	s	8.67	s (broadened peak)	8.68
**1″ (2H)**	t	2.29	t	2.29
**2″ (2H)**	m	1.46	m	1.46
**3″,4″ (4H)**	m	1.36–1.18	m	1.36–1.18
**5″ (3H)**	t	0.85	t	0.85

**Table 2:** Water solubility data obtained for pure CBD and its water-soluble glucosamine complexes.

CBD–Glucosamine Molar Ratio	CBD Concentration (mg/L)
pure CBD	below 0.06
1:1	21.1
1:2	26.5
1:3	28.0

**Figure A1:**
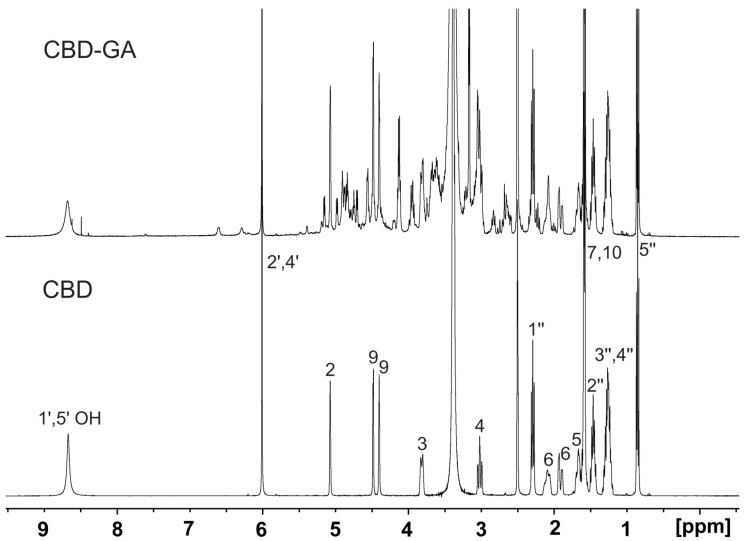
Part of ^1^H NMR spectra of samples of pure CBD and CBD-GA complex dissolved in 600 μL of DMSO-d6. Spectra were measured on 400 MHz NMR spectrometer at 25 °C. Assignment of protons of CBD is indicated in spectrum of pure CBD.

## Data Availability

Data available upon request.

## Abbreviations

The following abbreviations are used in this manuscript:

ATR	Attenuated total reflection
CAD	Charged aerosol detector
CBD	Cannabidiol
DMSO	Dimethyl sulfoxide
DSC	Differential scanning calorimetry
FL	Fluorescence
FTIR	Fourier transform infrared
GA	Glucosamine
HPLC	High-performance liquid chromatography
HSQC	Heteronuclear single quantum coherence
NMR	Nuclear magnetic resonance
MCT	Mercury cadmium telluride
PAMPA	Parallel artificial membrane permeability assay
PVDF	Polyvinylidene fluoride

## Appendix A

### Appendix A.1

The most intense IR bands identified from the ATR-FTIR spectra and rounded to 1 cm^−1^ precision are as follows.

For CBD: 3519, 3406, 2923, 2855, 1623, 1581, 1442, 1375, 1311, 1301, 1278, 1236, 1214, 1172, 1150, 1051, 1032, 1019, 1001, 961, 896, 882, 869, 861, 850, 827, and 804 cm^−1^.

For GA: 3353, 3270, 2921, 2878, 2690, 2663, 1587, 1456, 1415, 1355, 1317, 1259, 1071, 1016, 984, and 894 cm^−1^.

For the CBD-GA mixture: 3519, 3406, 3283, 2923, 2855, 2666, 1623, 1581, 1463, 1442, 1375, 1347, 1312, 1279, 1238, 1214, 1172, 1150, 1133, 1077, 1049, 1032, 1019, 1003, 961, 895, 882, 869, 861, 850, 827, and 804 cm^−1^.

For the CBD-GA complex: 3519, 3406, 3282, 2923, 2856, 2666, 1622, 1583, 1463, 1443, 1375, 1311, 1301, 1278, 1236, 1214, 1172, 1149, 1131, 1073, 1049, 1032, 1017, 1002, 983, 960, 895, 883, 869, 862, 850, 829, and 804 cm^−1^.

Underlined are the bands where differences are observed between the CBD-GA mixture and CBD-GA complex.

### Appendix A.2

The corresponding NMR data are as follows.

Pure CBD: ^1^H NMR (400 MHz, DMSO) δ 8.67 (s, 2H), 6.01 (s, 2H), 5.07 (s, 1H), 4.48 (d, *J* = 2.8 Hz, 1H), 4.39 (dd, *J* = 2.9, 1.5 Hz, 1H), 3.87–3.77 (m, 1H), 3.02 (m, 1H), 2.29 (t, *J* = 7.6 Hz, 2H), 2.16–2.02 (m, 1H), 1.91 (m, 1H), 1.66 (m, 2H), 1.59 (s, 3H), 1.57 (s, 3H), 1.46 (m, 2H), 1.36–1.18 (m, 4H), 0.85 (t, *J* = 7.0 Hz, 3H).

CBD-GA complex: ^1^H NMR (400 MHz, DMSO) δ 8.68 (s, 2H), 6.01 (s, 2H), 5.07 (s, 1H), 4.48 (d, *J* = 3.0 Hz, 2H), 4.39 (dd, *J* = 2.9, 1.6 Hz, 1H), 2.29 (t, *J* = 7.6 Hz, 2H), 1.91 (m, 1H), 1.66 (m, 2H), 1.59 (s, 3H), 1.57 (s, 3H), 1.46 (m, 2H), 1.36–1.18 (m, 4H), 0.85 (t, *J* = 7.0 Hz, 3H).

### Appendix A.3

The water eluates from glucosamine hydrochloride desalting were analysed in the undiluted form using a Vanquish HPLC system (Thermo Scientific) with CAD detection. This method is loosely based on the technical note published by Dolci et al. [38]. The column in use was Acclaim Trinity P1 (Thermo Scientific), with dimensions 100 mm × 2.1 mm, 3 µm particle size. The column flow rate was 0.25 mL/min at 30 °C. The elution was isocratic, with a mobile phase of 30% acetonitrile (*v*/*v*) in 10 mM ammonium acetate buffer pH 5. The injection volume was 2 µL, and the run time was 15 min. The CAD temperature was maintained at 40 °C.

### Appendix A.4

The concentration of CBD in dissolution and permeation test samples was assayed using an Agilent 1260 Infinity system with UV detection [39]. The column in use was Luna C18(2) (Phenomenex, Torrance, CA, USA), with dimensions 150 mm × 3 mm, 3 µm particle size. The column flow rate was 0.8 mL/min at 37 °C. The elution was isocratic, with the mobile phase consisting of acetonitrile–water at a ratio of 31:9 (*v*/*v*), with 10 mM ammonium formate and 0.1% (*v*/*v*) formic acid added (without pH adjustment). The injection volume was 5 µL, and the run time was 5 min. The UV detector wavelength was set at 275 nm.

### Appendix A.5

The concentration of atenolol and metoprolol in permeation test samples was assayed using a Vanquish HPLC system (Thermo Scientific) with FL detection [40]. The column in use was ACE Excel 2 C18 (Avantor, Radnor, PA, USA), with dimensions 100 mm × 2.1 mm, 2 µm particle size. The column flow rate was 0.2 mL/min at 35 °C. The elution was isocratic, with the mobile phase consisting of 50% methanol (*v*/*v*), with 0.1% (*v*/*v*) trifluoracetic acid added. The injection volume was 10 µL, and the run time was 4.5 min. The FL detector was set at 276 nm for excitation and 296 nm for emission.
